# Evaluation of larvicidal activity of the essential oil of *Allium macrostemon* Bunge and its selected major constituent compounds against *Aedes albopictus* (Diptera: Culicidae)

**DOI:** 10.1186/1756-3305-7-184

**Published:** 2014-04-15

**Authors:** Xin Chao Liu, Qiyong Liu, Ligang Zhou, Zhi Long Liu

**Affiliations:** 1 Department of Entomology, China Agricultural University, Haidian District, Beijing 100193, China; 2 State Key Laboratory for Infectious Disease Prevention and Control, National Institute for Communicable Disease Control and Prevention, Chinese Center for Disease Control and Prevention, Beijing 102206, China; 3 Department of Plant Pathology, China Agricultural University, Haidian District, Beijing 100193, China

**Keywords:** *Allium macrostemon*, *Aedes albopictus*, Larvicidal activity, Methyl propyl disulfide, Dimethyl trisulfide

## Abstract

**Background:**

During the screening programme for new agrochemicals from Chinese medicinal herbs and local wild plants, the essential oil of dried bulbs of *Allium macrostemon* Bunge (Liliaceae) was found to possess larvicidal activity against mosquitoes. The aim of this research was to determine the larvicidal activity of the essential oil and its major constituent compounds against the larvae of the Culicidae mosquito, *Aedes albopictus.*

**Methods:**

Essential oil of *A. macrostemon* was obtained by hydrodistillation and analyzed by gas chromatography (GC) and gas chromaotography-mass spectrometry (GC-MS). The activity of the essential oil and its two major constituents were evaluated, using World Health Organization (WHO) procedures, against the fourth instar larvae of *Ae. albopictus* for 24 h and larval mortalities were recorded at various essential oil/compound concentrations ranging from 9.0 - 150 μg/ml.

**Results:**

The essential oil of *A. macrostemon* exhibited larvicidal activity against the early fourth instar larvae of *Ae. albopictus* with an LC_50_ value of 72.86 μg/ml. The two constituent compounds, dimethyl trisulfide and methyl propyl disulfide possessed strong larvicidal activity against the early fourth instar larvae of *Ae. albopictus* with LC_50_ values of 36.36 μg/ml and 86.16 μg/ml, respectively.

**Conclusion:**

The results indicated that the essential oil of *A. macrostemon* and its major constituents have good potential as a source for natural larvicides.

## Background

Females of many species of mosquitoes consume blood from living vertebrates, including humans. They are an important public health concern around the world. In the process of feeding on blood, some of them transmit extremely harmful human diseases, such as yellow fever, dengue fever, malaria, several forms of encephalitis, filariasis and chikungunya [1]. Millions of deaths occur globally each year due to mosquito-borne diseases with a disproportionate effect on children and elders in developing countries [2]. Mosquitoes also cause allergic responses on humans that include local skin and systemic reactions such as angioedema [3]. The yellow fever mosquito (*Aedes aegypti* L.) and the Asian tiger mosquito (*Ae. albopictus* Skuse) are the two main species of mosquito responsible for dengue fever and malaria in China [4]. Mosquito larvae and pupae are currently controlled by the usage of synthetic chemical insecticides organophosphates (e.g. temephos, fenthion, malathion), and insect growth regulators (e.g. diflubenzuron, methoprene). Their repeated use has disrupted the natural biological control systems, sometimes resulting in the widespread development of resistance as well as undesirable effects on non-target organisms, toxic residues in food, workers’ safety, and high cost of procurement [5]. These problems have warranted the need for developing alternative strategies using ecofriendly products. From this point of view, botanical pesticides, including essential oils, are promising since they are effective, environmentally friendly, easily biodegradable and often inexpensive. Moreover, herbal sources provide a lead for discovering new insecticides [5]. It is suggested that many essential oils and constituent compounds derived from various essential oils can exert toxic activity against mosquito species [6-11]. During our mass screening program for new agrochemicals from wild plants and Chinese medicinal herbs, essential oil of dried bulbs of *Allium macrostemon* Bunge (Family: Liliaceae) was found to possess larvicidal activity against the Asian tiger mosquito, *Ae. albopictus*.

The dried bulbs of *A. macrostemon* are well known as the traditional Chinese medicine “Xie Bai”, which is used for the treatment of thoracic pain, stenocardia, heart asthma and diarrhea [12]. The bulbs and leaves, eaten as part of the diet, are emmenagogue, nervine and tonic. When added to the diet on a regular basis, they help to reduce blood cholesterol levels, act as a tonic to the digestive system and also tonify the circulatory system [12]. In the previous reports, various furostanol saponins, steroidal saponins and spirostane saponins have been isolated and identified from the plant [13-26]. Chemical composition of essential oil of *A. macrostemon* has been analyzed previously [27-29]. Juice of this plant is used as a moth repellent [13]. However, a literature survey has shown that there is no report on insecticidal activity of the essential oil of *A. macrostemon* and its constituent compounds against mosquitoes, thus we decided to investigate the larvicidal activity of the essential oil of *A. macrostemon* and its major constituents against the Asian tiger mosquito.

## Methods

### Plant material

Air-dried bulbs of wild *A. macrostemon* (5 Kg, collected during August, 2012, from Baicheng city, Jilin Province, 44.57°N latitude and 121.50° E longitude) were purchased from the Anguo Medicinal Herb Market, Hebei 071200, China. The plant sample was identified by Dr Liu QR (College of Life Sciences, Beijing Normal University, Beijing 100875, China) and a voucher specimen (CMH-XieBai-Liaoning-2012-06) was deposited at the Department of Entomology, China Agricultural University, Beijing, China. The sample was ground to powder using a grinding mill (Retsch Muhle, Haan, Germany) and was subjected to hydrodistillation using a modified Clevenger-type apparatus for 6 h and extracted with *n*-hexane. Anhydrous sodium sulphate was used to remove water after extraction. Essential oils were stored in airtight containers in a refrigerator at 4°C for subsequent experiments.

### Gas chromatography–mass spectrometry (GC-MS)

Profiles of volatile constituents were determined using an Agilent 5973 GC-MS system operating in the EI mode at 70 eV [equipped with a 30 m HP-5MS column (0.25 mm × 30 m × 0.25 μm) and coated with 5% phenyl-methylpolysiloxane using a HP-5MS (df = 0.25 μm) (Agilent J&W Scientific, USA)]. The temperature program used for the analysis was as follows: initial temperature at 60°C, held for 1 min, ramped at 4°C/min to 290°C and held for 0.5 min. Helium was the carrier gas at 1.0 ml/min; the sample (1 μl 1/100, v/v, in acetone) was injected in the split mode (1:10). The injector and detector temperatures were preformed at 230°C and 300°C, respectively. Most constituents were identified by gas chromatography by comparison of their retention indices with those of the literature or with those of authentic compounds available in our laboratories. The retention indices were determined in relation to a homologous series of *n*-alkanes (C_8_-C_24_) under the same operating conditions. Further identification was made by comparison of their mass spectra with those stored in NIST 05 and Wiley 275 libraries or with mass spectra from literature [30]. Component relative percentages were calculated based on normalization method without using correction factors.

### Insect cultures and rearing conditions

Mosquito eggs of *Ae. albopictus* utilized in bioassays were obtained from a laboratory colony maintained in the Department of Vector Biology and Control, Institute for Infectious Disease Control and Prevention, Chinese Center for Disease Control and Prevention. The dehydrated eggs were put into plastic trays containing tap water to hatch and yeast pellets served as food for the emerging larvae. The egg batches, collected daily, were kept wet for 24 h and then placed in mineral water in the laboratory at 26-28°C under natural summer photoperiod for hatching. The newly emerged larvae were then isolated in groups of ten specimens in 100 ml tubes with mineral water and a small amount of dog food. Larvae were controlled daily until they reached the fourth instar, when they were utilized for bioassays (within 12 h).

### Bioassays

Range-finding studies were run to determine the appropriate testing concentrations. Concentrations of 150.0, 75.0, 37.5, 18.5, and 9.0 μg/ml of the crude extract/compound were tested. The larval mortality bioassays were carried out according to the test method of larval susceptibility as suggested by the World Health Organization [31]. Twenty larvae were placed in glass beaker with 250 ml of aqueous suspension of test material at various concentrations and an emulsifier (DMSO) was added in the final test solution (less than 0.05%). Five replicates were run simultaneously per concentration and with each experiment, a set of controls using 0.05% DMSO and untreated sets of larvae in tap water, were also run for comparison. The two constituents, dimethyl trisulfide (98%) and methyl propyl disulfide (97%) were purchased from Aladdin Industrial Inc. (Shanghai, China). For comparison, commercial chlorpyrifos [purchased from National Center of Pesticide Standards (8 Shenliao West Road, Tiexi District, Shenyang 110021, China)] was used as a positive control. The toxicity of chlorpyrifos was determined at 5, 2.5, 1.25, 0.6, and 0.3 μg/ml. The assays were placed in a growth chamber (L16:D9, 26-27°C, 78-80% relative humidity). Mortality was recorded after 24 h of exposure and the larvae were starved within this period. The percentage of mortality was corrected for control mortality using Abbott’s formula. Results from all replicates for the pure compounds/oil were subjected to probit analysis using the PriProbit Program V1.6.3 to determine LC_50_ values and their 95% confidence intervals [32].

## Results and discussion

### Essential oil chemical composition

The yield of yellow essential oil of *A. macrostemon* was 0.56% (v/w based on dry weight) while its density was 0.92 g/ml. A total of 16 components from the essential oil of *A. macrostemon* were identified, accounting for 98.27% of the total oil. The principal constituents of *A. macrostemon* essential oil were methyl propyl disulfide (47.2%) and dimethyl trisulfide (37.2%) followed by diallyl thiosulfinate (3.3%) and 1,3-dithiane (2.6%) (Table 1). Monoterpenoids only represented 3 of the 16 compounds, corresponding to only 0.29% of the whole oil, while 13 of the 16 constituents were S-containing compounds (97.98% of the crude essential oil). A large variation in the chemical profiles of the *A. macrostemon* essential oil in this study was observed from those of previous reports [27-29]. For example, dimethyl trisulfide (21.0%), methyl propyl trisulfide (18.6%), methyl propyl disulfide (17.0%) and isopropyl propyl disulfide (10.6%) were the major compounds in the essential oil of *A. macrostemon* harvested from Shenyang, Liaoning province, China [27]. However, the essential oil of *A. macrostemon* collected from Jilin province, China mainly contained dimethyl trisulfide (29.98%), methyl allyl trisulfide (29.01%), methyl allyl disulfide (6.87%), and methyl propyl trisulfide (5.74%) [28], while the essential oil of *A. macrostemon* obtained from Sichuan province, China contained methyl allyl trisulfide (20.73%), dimethyl trisulfide (16.01%), dimethyl tetrasulfide (9.25%), and dimethyl disulfide (5.62%) [29]. The results above suggest that there are great variations in chemical composition of essential oil of *A. macrostemon*. This variation could be due to analytic methods, population, different parts used, harvest time and local, climatic and seasonal factors as well as storage duration of medicinal herb. Thus, further studies on plant cultivation and essential oil standardization are needed.

**Table 1:** **Chemical constituents of the essential oil of *Allium macrostemon***

**Peak no.**	**Compound**	**RI**^**a**^	**RI**^**b**^	**Content (%)**
1	Diallyl sulfide^*^	848	848	0.34
2	Allyl isothiocyanate	890	890	0.51
3	Methyl allyl disulfide^*^	915	912	1.58
4	α-Pinene^*^	937	939	0.14
5	Methyl propyl disulfide^*^	950	946	47.23
6	Dimethyl trisulfide^*^	975	972	37.18
7	β-Pinene^*^	980	979	0.08
8	1,3-Dithiane	1027	1027	2.57
9	Limonene^*^	1029	1024	0.07
10	Diallyl disulfide^*^	1077	1076	1.52
11	Methyl allyl trisulfide^*^	1134	1133	0.68
12	Methyl propyl trisulfide^*^	1168	1168	1.27
13	Dimethyl tetrasulfide	1214	1212	0.59
14	Diallyl trisulfide^*^	1296	1289	0.52
15	Diallyl thiosulfinate	1325	1324	3.29
16	Allyl methyl tetrasulfide	1386	1384	0.82
	Total identified			98.39
	Monoterpenoids			0.29
	S-Containing compounds			98.10

^*^Identification by co-injection of authentic compounds; ^a^RI, retention index as determined on a HP-5MS column using a homologous series of *n*-hydrocarbons; ^b^RI, retention index reported in the literature [30,33].

### Larvicidal activity

The essential oil of *A. macrostemon* exhibited larvicidal activity against the early fourth instar larvae of *Ae. albopictus* with an LC_50_ value of 72.86 μg/ml (Table 2). The two constituent compounds, dimethyl trisulfide and methyl propyl disulfide possessed strong larvicidal activity against the early fourth instar larvae of *Ae. albopictus* with LC_50_ values of 36.36 μg/ml and 86.16 μg/ml, respectively (Table 2). The commercial insecticide, chlorpyrifos showed larvicidal activity against the mosquitoes with a LC_50_ value of 1.86 μg/ml, thus the essential oil of *A. macrostemon* was 39 times less toxic to *Ae. albopictus* larvae compared with chlorpyrifos. However, compared with the other essential oils/extracts in the literature, essential oil of *A. macrostemon* exhibited stronger or the same level of larvicidal activity against *Ae. albopictus* larvae, e.g. essential oils of *Eucalyptus urophylla* (LC_50_ = 95.5 μg/ml) [34]; essential oils of *Achillea millefolium* (LC_50_ = 211.3 μg/ml), *Helichrysum italicum* (LC_50_ = 178.1 μg/ml), *Foeniculum vulgare* (LC_50_ = 142.9 μg/ml) [35]; ethanol extract from *Cryptomeria japonica* (LC_50_ = 93.8 μg/ml) [36], and ethanol extract of *Knema attenuata* (LC_50_ = 141 ppm) [37] as well as a solution of 80% ethanol extract of fresh garlic (*Allium sativum*) [38]. Among the two constituent compounds, methyl propyl disulfide exhibited almost the same level of larvicidal activity as the essential oil and dimethyl trisulfide possessed stronger larvicidal activity than the crude oil against *Ae. albopictus* larvae. Moreover, compared with chlorpyrifos, dimethyl trisulfide exhibited 19 times less toxicity against *Ae. albopictus* larvae (Table 2).

**Table 2:** **Larvicidal activity of the essential oil of *Allium macrostemon *** **and its major constituents against fourth-instar larvae of *Aedes albopictus***

**Treatment**	**LC**_**50 **_**(μg/ml)**	**LC**_**95 **_**(μg/ml)**	**Slope ± SD**	**Chi-square value**
	**(95% CL)**	**(95% CL)**		
Essential oil	72.86	132.84	2.72 ± 0.27	7.73*
	(66.02-78.67)	(126.76-145.17)		
Dimethyl trisulfide	36.36	55.65	2.61 ± 0.26	10.60*
	(33.23-39.64)	(49.43-61.27)		
Methyl propyl disulfide	86.16	144.16	2.59 ± 0.25	8.94*
	(79.53-91.43)	(130.22-155.82)		
Chlorpyrifos	1.86	6.65	0.87 ± 0.01	3.13*
	(1.71-2.05)	(6.21-7.48)		

*Significant at *P* < 0.05 level.

In the previous reports, the two constituents exhibited antimicrobial activities against several bacteria and fungi as well as nematodes [39-42]. There are numerous reports on insecticidal activity of sulfide-containing compounds against pests including the two constituent compounds. For example, dimethyl trisulfide was demonstrated to exhibit toxicity to African cowpea bruchid (*Bruchidius atrolineatus*) [43]. Dimethyl trisulfide and methyl propyl disulfide also exhibited larvicidal activity against the yellow fever mosquito (*Ae. aegypti*) [44,45]. The above findings suggest that the essential oil and the two major constituent compounds show the potential to be developed as possible natural larvicides for the control of mosquitoes.

It seems that this plant is quite safe for human consumption because it has been used as a common vegetable or spice in Chinese food [46] and also as a medicinal herb for the treatment of thoracic pain, stenocardia, heart asthma and diarrhea [12]. Dimethyl trisulfide and methyl propyl disulfide can be used as food additives and flavours in China. However, no information on toxicity of the essential oil and the isolated constituents to humans are available. Thus, to develop a practical application for the essential oil and its constituents as novel insecticides, further research into the safety of the essential oil/compounds to humans is needed. Additional studies on the development of formulations are also necessary to improve the efficacy and stability and to reduce costs. Moreover, field evaluation and further investigations on the effects of the essential oil and its constituent compounds on non-target organisms are necessary.

## Conclusions

The essential oil of *Allium macrostemon* bulbs and its two major constituents demonstrate strong larvicidal activity against *Aedes albopictus* mosquito larva. Our results suggested that the essential oil of *A. macrostemon* and the two major constituents may be recommended effectively in mosquito control program but needs to be further evaluated for safety in humans and to enhance their activity.

## Competing interests

The authors declare that they have no competing interests.

## Authors’ contributions

ZLL designed the work and supervised the manuscript. XCL, QL and LZ performed experiments, interpretation of data and drafted the manuscript. All authors read and approved the final version of the manuscript.
